# Curvature increases permeability of the plasma membrane for ions, water and the anti-cancer drugs cisplatin and gemcitabine

**DOI:** 10.1038/s41598-019-53952-2

**Published:** 2019-11-20

**Authors:** Semen Yesylevskyy, Timothée Rivel, Christophe Ramseyer

**Affiliations:** 1 Laboratoire Chrono Environnement UMR CNRS 6249, Université de Bourgogne Franche-Comté, 16 route de Gray, 25030 Besançon, Cedex France; 2 Department of Physics of Biological Systems, Institute of Physics of the National Academy of Sciences of Ukraine, Prospect Nauky 46, 03028 Kyiv, Ukraine

**Keywords:** Computational biophysics, Membrane structure and assembly, Supramolecular assembly, Drug delivery

## Abstract

In this work the permeability of a model asymmetric plasma membrane, for ions, water and the anti-cancer drugs cisplatin and gemcitabine is studied by means of all-atom molecular dynamics simulations. It is shown for the first time that permeability of the highly curved membrane increases from one to three orders of magnitude upon membrane bending depending on the compound and the sign of curvature. Our results suggest that the membrane curvature could be an important factor of drug translocation through the membrane.

## Introduction

Passive permeability of cell membranes is of great importance for pharmacology since many drugs are known to permeate spontaneously through the plasma membrane of the target cells. The membranes of real cells are highly heterogeneous and possess regions with very different curvature – from essentially flat to highly curved, such as filopodia, cilia, membrane blebs, caveolae, etc^1^. Origins of the membrane curvature are diverse and range from spontaneous curvature of the lipids^2–4^ to the influence of membrane proteins and cytoskeleton^5^. Membrane curvature is intrinsically connected to the asymmetry of lipid^6,7^ and cholesterol^8–10^ content of membrane monolayers.

The shape of the curved membrane surface is described by the mean and Gaussian curvatures^11^. Mean curvature measures an extent of the average membrane bending, while the Gaussian curvature determines the topological type of the surface (sphere-like, cylinder-like, or saddle-like). In the sake of simplicity in this paper we concentrate on the mean curvature in the membrane with cylindrical geometry, which is bent in a single plane.

Up to now it is not known which regions of the membrane are preferred for drug permeation and whether such preference exists. Moreover, membrane morphologies of normal and malignant cells differ substantially^12,13^. Malignant cells could have more ragged surface with many membrane blebs and protrusions or, in contrast, be round and featureless in comparison to their normal counterparts. It is not known whether these differences in membrane morphology affect uptake of the drugs by cancer cells.

Being a transient dynamic property of the membrane, which changes in time and in different sites of the cell surface, the curvature is difficult to study experimentally. Particularly studying the influence of curvature on the passive transport of small molecules and drugs through the membrane remains challenging.

Molecular dynamics (MD) simulations allow overcoming these difficulties by providing atomistic view of the membrane in completely controllable environment. Recent MD study revealed significant and non-trivial effects of curvature on different membrane properties such as distribution of cholesterol, area per lipid, thickness of the leaflets and the order parameter of the lipid tails^14,15^. It is plausible that such pronounced curvature-dependent changes in the lipid packing and cholesterol distribution should affect the permeability of the membrane for hydrophilic compounds. However, it is hard to deduce whether permeability increases of decreases upon membrane bending and to what extent.

MD simulations provide unique opportunity of studying diffusion of drugs and small molecules through realistic model membranes in atomic details but, to the best of our knowledge, all existing studies were performed on the planar bilayers only. In order to study the influence of curvature on permeability one has to sample the transmembrane diffusion of the ligand in the parts of the membrane with desired curvature only. There are two ways of achieving this: either by sampling large membrane patch for a long time and selecting the regions with needed curvature, utilizing one of known methods of determining local^16^ or global^17^ membrane curvatures, or by maintaining the curvature of the membrane around desired value by artificial restrains. The first approach, while being closer to reality, is currently well beyond the time scales available for all-atom MD simulations. In contrast, the second approach is currently computationally tractable. In our previous work^14^ we developed an efficient technique of restricting global membrane curvature to any desired value, which does not affect fine-grained dynamics of membrane components and key macroscopic membrane properties.

**Figure 1:**
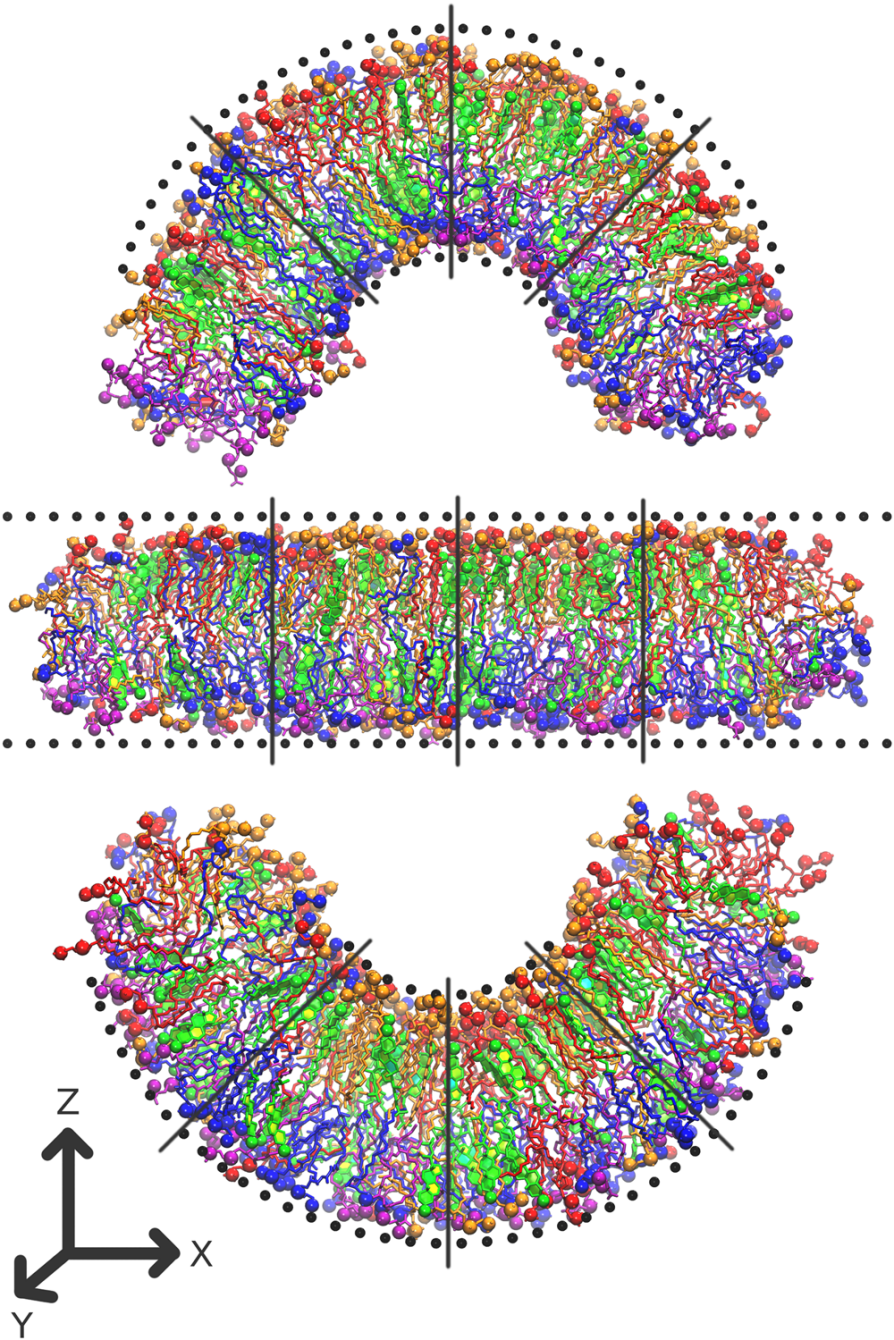
Simulated systems with the curvatures 0.2 nm^−1^ (top panel), 0.0 nm^−1^ (middle panel) and −0.2 nm^−1^ (bottom panel). Outer membrane leaflet is on top. PC is red, PE is blue, PS is violet, SM is orange and cholesterol is green (see Methods for definitions of abbreviations). N and P atoms of the lipid head group and the hydroxyl oxygen of cholesterol are shown as spheres. Black spheres show dummy particles which maintain the membrane shape. Black lines show approximate axes where the ligands are restrained during umbrella sampling simulations. Water molecules and ions are not shown for clarity.

In this work we used all-atom MD simulations for determining permeability of highly curved membranes for water, ions and small hydrophilic anti-cancer drugs cisplatin and gemcitabine. The usage of high curvature allows detecting curvature-related changes of permeability reliably and in reasonable computational time, however the magnitude of these effects may differ from ones observed in real cells (see Discussion for details). We used a model of mammalian plasma membrane with asymmetric lipid composition and 33% mole fraction of cholesterol. We performed simulations for three values of curvature: 0 nm^−1^ (flat membrane), 0.2 nm^−1^ (outer monolayer is convex) and −0.2 nm^−1^ (inner monolayer is convex) as shown in Fig. 1. The potentials of mean force (PMFs), diffusion coefficients (*D*) and permeation resistances (*R*) across the membrane were computed. The dependence of permeability (*P*) on the curvature for all studied ligands is estimated.

## Results

**Figure 2:**
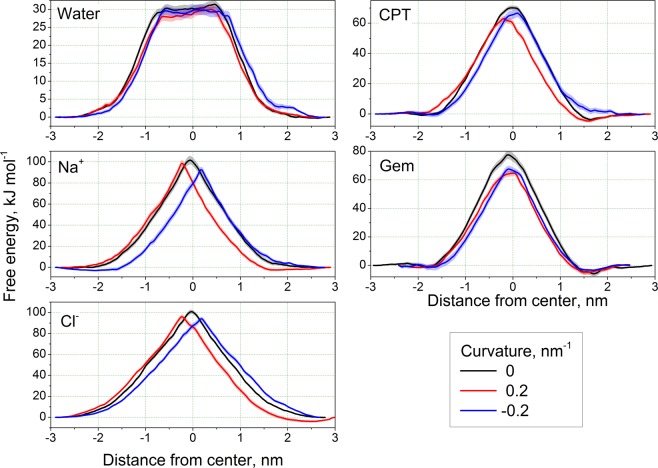
Potentials of mean force for studied ligands. The error bars are shown as semi-transparent bands.

Analysis of the PMFs shows that the energy barriers for all permeating ligands are lower in the curved membrane in comparison to the flat one (Fig. 2) while the magnitude of this effect varies between the ligands (Table 1).

**Table 1:** Maximal heights of the energy barriers (kJ/mol).

Curvature	Na^+^	Cl^-^	Water	CPT	Gem
0	101.1	101.6	31.4	70.1	77.5
0.2	98.3 (0.97)	96.4 (0.95)	30.2 (0.96)	63.1 (0.90)	64.8 (0.83)
−0.2	92.7 (0.92)	93.4 (0.92)	29.9 (0.95)	66.7 (0.95)	67.6 (0.87)

Values in parentheses show relative height of the barrier with respect to the flat membrane for particular ligand.

The peaks of the PMFs shift systematically towards concave monolayer (Fig. S1, see Supplementary Information for definition of the peak position). Such shift is expected since the convex monolayer has larger area per lipid and looser packing of the lipids than the flat membrane while the concave monolayer is more densely packed^14^ and imposes higher barrier on the permeant.

**Figure 3:**
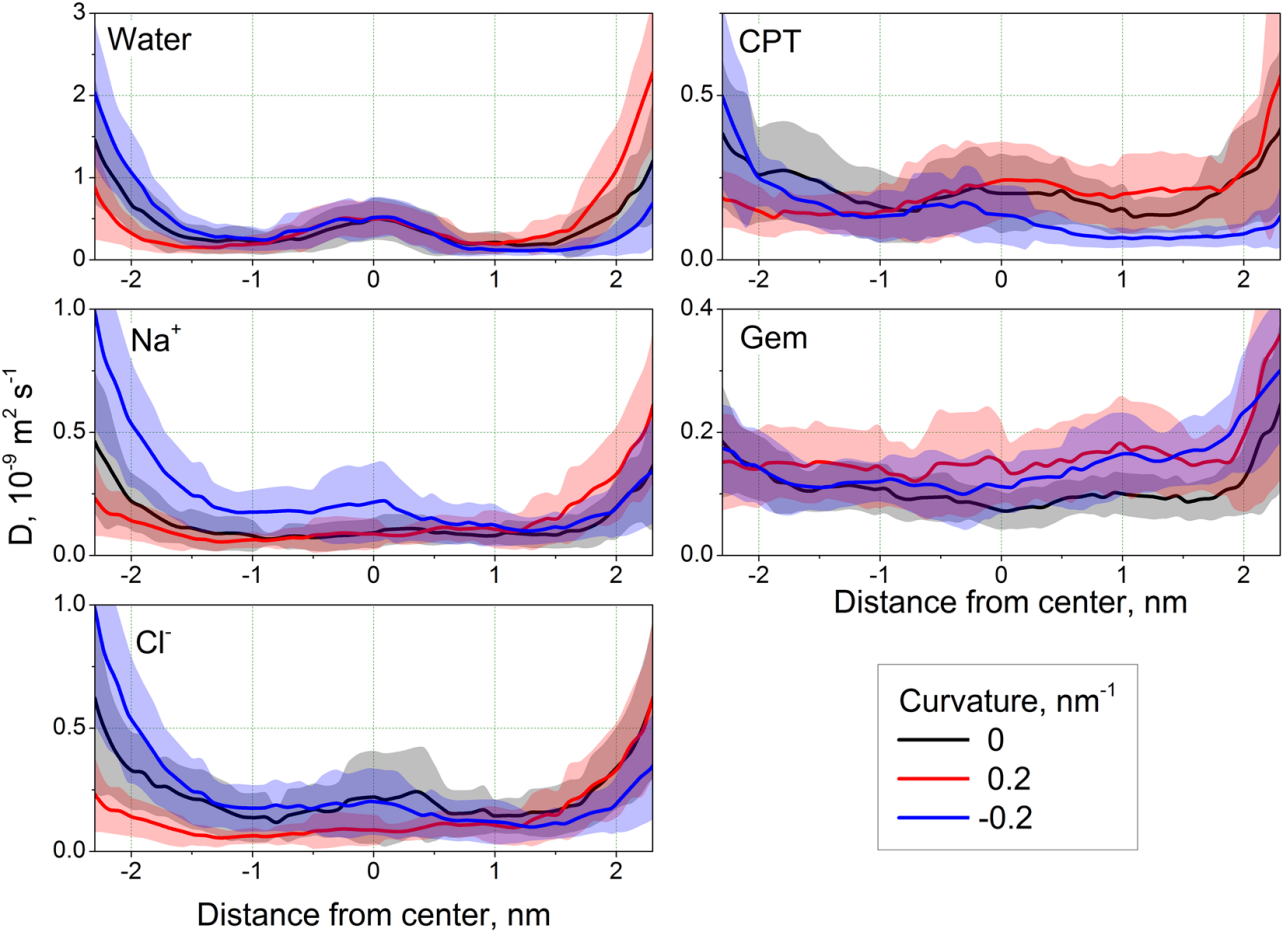
Diffusion coefficients of the studied ligands. The error bars are shown as semi-transparent bands.

The diffusion coefficients of the ligands change consistently upon the membrane bending (Fig. 3). For all ligands except Gem the *D* is lower for the concave monolayer in comparison to the flat membrane which reflects more congested environment in this monolayer. In the case of Gem the changes of *D* are small and do not allow to deduce obvious trend from them.

The absolute values of *D* do not change much upon bending within 1 nm from the center of the membrane. The maximal change in this area is about 1.5 times for all ligands. Much larger variations are observed in the regions of lipid head groups and bulk solvent. In order to estimate the influence of these regions on total permeability we analyzed the membrane resistance *R* (Fig. S2). For all studied ligands resistance drops by several orders of magnitude at the distance of ~1 nm from the membrane center (note the log scale in Fig. S2) thus the changes of *D* outside this region has almost no effect on permeability. This suggests that the permeability will depend only slightly on *D* and the shape and height of the PMF plays the major role.

Permeability coefficients *P* of the ligands are summarized in Table 2. There is a systematic trend, which is consistent with the changes of PMFs upon bending. The *P* of all ligands increases upon membrane bending. For Na^+^ and Cl^−^ ions this increase is much larger for negative curvature (when inner monolayer in convex). For water the effect is comparable for positive and negative curvatures. For CPT and Gem the decrease of *P* is much larger for positive curvature (when inner monolayer in concave). Comparison with Table 1 shows that the permeability follows exactly the same trend as the decrease of the energy barrier observed on PMFs in curved membranes.

**Table 2:** Permeability coefficients (m∙s^−1^) for all studied ligands.

Curvature, nm^−1^	0	0.2	−0.2
Water	2.5∙10^−6^ ± 3.6∙10^−8^	5.5∙10^−6^ ± 7.2∙10^−8^ (2.1)	3.7∙10^−6^ ± 5.3∙10^−8^ (1.5)
Na^+^	1.5∙10^−17^ ± 1.2∙10^−18^	8.4∙10^−17^ ± 6.9∙10^−18^ (5.3)	1.7∙10^−15^ ± 1.3∙10^−16^ (107.5)
Cl^-^	4.6∙10^−17^ ± 2.4∙10^−18^	1.5∙10^−16^ ± 8.7∙10^−18^ (3.2)	6.4∙10^−16^ ± 3.3∙10^−17^ (13.8)
CPT	2.3∙10^−12^ ± 6.2∙10^−14^	5.0∙10^−11^ ± 1.6∙10^−12^ (22.1)	7.0∙10^−12^ ± 3.1∙10^−13^ (3.0)
Gem	8.3∙10^−14^ ± 3.6∙10^−15^	1.4∙10^−11^ ± 4.2∙10^−13^ (164.5)	4.5∙10^−12^ ± 1.5∙10^−13^ (53.7)

Numbers in parentheses show the ratio of permeability in comparison to zero curvature for particular ligand.

There is no obvious dependence of the absolute change of permeability on the size or chemical nature of the ligand. For example, the absolute change for Na^+^ ions is one order of magnitude larger than for Cl^−^ ions for positive curvature, but they are of the same order of magnitude for the negative curvature.

## Discussion

The main message which could be inferred from our data is that the membrane curvature increases permeability of the membranes with asymmetric lipid content for water, ions and anti-cancer drugs cisplatin and gemcitabine. To the best of our knowledge this is the first report of the curvature dependence of membrane permeability and the first dedicated simulation setup, which allows studying this phenomenon. Since this work is pioneering in the field, our first goal was to demonstrate an existence of the effect of curvature on passive transport of various hydrophilic compounds. The value of curvature used in this work is probably larger than typically observed in real membranes (see discussion below) but it makes simulations computationally tractable, clearly demonstrates an existence of the effect and its sign. It also provides a solid reference for the future studies, where the effects could be more subtle and harder to interpret due to smaller curvatures.

We observed increase of permeability up to three orders of magnitude depending on the direction of bending and the permeating ligand. Particularly, the difference in permeability in respect to the flat membrane reaches ~160 times for gemcitabine and ~22 times for cisplatin. Even if this effect would be weaker in real cells with smaller membrane curvature it allow us speculating that the most of passive transport of these drugs is likely to occur in highly curved regions of their membrane. It also suggests that the changes of membrane curvature in malignant cells may contribute to the drug uptake and resistance, which opens new perspectives for further research and possible practical applications.

### The note on the magnitude of membrane curvature

The absolute values of the plasma membrane curvature in real cells are rarely reported in the literature thus most of the data in this domain come from model experiments and numerical simulations. The curvatures of 0.01–0.035 nm^−1^ are reported for invaginations induced by the protein toxins^18,19^, viral capsid proteins^20^ and annexins^21^. F-BAR domain proteins may induce curvature up to 0.05–0.1 nm^−1 ^^22^. Synaptic vesicles and cell organelles are known to have the curvature up to 0.066 nm^−1 ^^23,24^. Even larger curvatures seem to appear in the membranes transiently. The undulation spectrum of the model lipid bilayers extend to the short wavelengths corresponding to transient curvatures as large as 0.1–0.2 nm^−1^ both in all-atom^25^ and coarse-grained simulations^26^. Significantly curved membrane structures may also form spontaneously in highly asymmetric bilayers^17^ and during membrane remodeling, such as fusion and vesicle budding^1,27^.

The most typical radii of curvature in the range of hundreds of nm are hard to access in atomistic MD simulations due to very large size of the simulation box and small magnitudes of all curvature-related effects, which require prohibitively large simulation times. The extreme curvatures of 0.1–0.2 nm^−1^ seems to be less common in real cells but still of significant interest. In addition to their possible direct role in cellular phenomena high curvatures provide an excellent reference point at which all curvature-related effects are clearly visible. In this work we used large curvature of 0.2 nm^−1^ which is unlikely to be commonly observed in real membranes but makes all-atom simulations tractable and allows to detect curvature-related changes of permeability with good confidence. This is especially important providing current limitations of the MD methodology of computing permeabilities, described below and in the Supporting Information. Large uncertainties in computed PMFs and diffusion coefficients could make detection of the subtle effects of smaller realistic curvatures problematic without prohibitive computational efforts.

### Methodology of computing permeability and its limitations

In our study we rely on pragmatic usage of the inhomogeneous solubility-diffusion (ISD) model^28,29^. We are aware about serious limitations of this approach and never compare different ligands with each other in terms of absolute values of permeability or translocation energy barriers. However, we compare the values for the same ligand in the same membrane but with different curvatures. Any systematic errors should be very similar in such simulations, which makes them directly comparable to each other (see detailed discussion in Supplementary Information).

The curved membranes used in this study were validated extensively in our previous works^14,30^. It was demonstrated that the dummy particles, which maintain the membrane shape, have minimal influence on the membrane structure and dynamics of individual lipids^14^. It was also shown that the PMFs of multiple permeating ligands located in different regions of complex multicomponent membrane provide sufficient sampling for making reliable semi-quantitative conclusions^30^. That is why we are convinced that obtained trends are robust and reflect real influence of the membrane curvature on its permeability for small hydrophilic compounds and drugs. We are aware that the magnitude of these effects at high curvature of 0.2 nm^−1^ is likely to be larger than at moderate curvatures of 0.01–0.05 nm^−1^ commonly observed in real membranes. Thus obtained results should not be treated as quantitative for real cells.

### Future directions

To our knowledge this work is the first report of the curvature-dependent permeability of the hydrophilic compounds in the lipid membranes with realistic lipid composition. Our simulations reveal this effect clearly but currently shed no light on its molecular mechanism. Future studies should concentrate on the exact role of different lipid species and cholesterol content on the permeability in curved membranes. More realistic curvatures of 0.01–0.05 nm^−1^ should be used to make the results closer to typical membranes of real cells, which require further methodological development to make such simulations computationally tractable.

## Materials and Methods

All simulations were performed using GROMACS software^31^, versions 5.1.2 and 2018.2. The slipids force field^32^ is used for lipids in combination with AMBER99sb force field for water and the ions. All simulations were performed in NPT conditions with Parrinello-Rahman barostat at 1 atm and velocity rescale thermostat^33^ at 320 K. Such temperature ensures liquid state of our cholesterol-rich membrane, speeds up equilibration of the system and enhances sampling. The group cutoff scheme was used^34^. An integration step of 1 fs was used for cisplatin (as required by its flexible topology^35^) and 2 fs for all other ligands. For simulations with 2 fs time step all bonds were converted to rigid constraints. Long range electrostatics was computed with the PME method^36^. Preparation of the systems and data analysis was performed with Pteros 2.0 molecular modeling library^37,38^. VMD 1.9.3 was used for visualization^39^.

Topologies of water and ions were taken from AMBER99sb force field. Topologies of cisplatin and gemcitabine were developed and tested in our previous works^35,40^ respectively. The QDSol topology of cisplatin was used^35^.

The simulation setup for curved asymmetric membranes developed in our previous works^14,41^ was used. The membrane is prepared as a bicelle which is limited by semi-cylindrical caps in XZ plane and forms an infinite bilayer in Y direction (Fig. 1). When the curvature is imposed on the membrane the areas of concave and convex monolayers become different and the lipids redistribute through the caps of the bicelle in order to compensate for resulting mechanical strain. The caps serve as “compensating reservoirs” which store excessive lipids from concave monolayer and donate the lipids to convex monolayer. This setup has shown robust performance in both coarse-grained and atomistic simulations^14,41^.

Table 3 shows the lipid content of the monolayers designed according to well-established lipid content of mammalian erythrocyte membranes^42^. Phosphatidylinositol was not included into simulations due to its small concentration, which results in about one molecule per system.

**Table 3:** Lipid content (absolute number of molecules) of the monolayers.

Component	Outer monolayer	Inner monolayer
SM (sphingomyelin)	42	12
PC (1,2-dioleoyl-sn-glycero-3-phosphocholine)	46	14
PE (1,2-dioleoyl-sn-glycero-3-phosphoethanolamine)	14	46
PS (1,2-dioleoyl-sn-glycero-3-phospho-L-serine)	0	30
Cholesterol	51	51

Initial cholesterol distribution is shown.

The mixing of the lipids from different monolayers in the regions of bicelle caps is prevented by imposing selective artificial repulsive potentials developed in our previous works^14,16,41,43^.

We use the method of keeping global membrane curvature at given value by shaping the membrane by means of dummy particles^14^. In brief the idea is to put the membrane between two repulsive surfaces of artificial particles (the walls) which scaffold the global shape of membrane but do not affect lateral dynamics of individual lipids.

The system is pre-equilibrated as a bilayer with periodic boundary conditions for 10 ns and then converted to a bicelle by adding extra layers of water from both sides in X direction. After that the restricting walls are added and the system is equilibrated as a planar bicelle for ~250 ns. After that the bending procedure was applied by moving the wall particles gradually as described in details in Supplementary Information. The systems with the curvatures of 0.2 nm^−1^ and −0.2 nm^−1^ (Fig. 1) were used for production simulations.

The inhomogeneous diffusion (ISD) model was used to compute permeabilities of the ligands. The umbrella sampling simulations were used to compute the potentials of means force and the diffusion coefficients of the ligands across the membrane.

The details for all the steps of system preparation, simulation, analysis and error estimation are provided in Supplementary Information.

## Conclusions

It is shown that permeability of the asymmetric lipid membrane, which mimics the composition of mammalian plasma membrane, for ions, water, cisplatin and gemcitabine strongly depends on the curvature. Highly curved membrane with the curvature of 0.2 nm^−1^ is one to three orders of magnitude more permeable for ions water, cisplatin and gemcitabine in comparison to flat one depending on the permeating compound and the sign of curvature. Our results show that high membrane curvature should not be neglected during evaluation of the membrane permeability for hydrophilic drugs. Further research is necessary to elucidate the role of membrane curvature modulation in cancer cells in the mechanisms of drug uptake and resistance due to curvature-dependent permeability of the common anti-cancer drugs.

## Supplementary information

Supplementary information
